# Research Progress in Single-herb Chinese Medicine and Compound Medicine for Knee Osteoarthritis

**DOI:** 10.2174/0113862073264850231116055745

**Published:** 2024-01-04

**Authors:** Guanghui Zhou, Xianquan Zhang, Zhuoxu Gu, Jinlong Zhao, Minghui Luo

**Affiliations:** 1 The Second Clinical College of Guangzhou University of Chinese Medicine, Guangzhou, 510405, China;; 2 The Second Affiliated Hospital of Guangzhou University of Chinese Medicine, Guangzhou, 510120, China

**Keywords:** Knee, chinese medicine, single herb, compound, osteoarthritis, signaling pathways, research progress, OA

## Abstract

Due to an increase in the aging population, osteoarthritis (OA), especially knee osteoarthritis (KOA), has increasingly become one of the diseases affecting the quality of life of the elderly. As the pathogenesis of KOA is still unclear, Western medicine treatment lacks specificity, and surgical treatment is difficult to cover all patients. Therefore, in recent years, traditional Chinese medicine (TCM) for the conservative treatment of KOA has received increasing attention. The advantages of TCM are clear, such as relief of symptoms, fewer adverse reactions, and wider applicability to patients. This paper mainly discusses the research progress in single-herb TCM and TCM compounds for KOA, aiming to demonstrate the effectiveness of TCM in the treatment of KOA. It also provides ideas for future research on the treatment of KOA by TCM and the pathogenesis of knee osteoarthritis.

## 1 INTRODUCTION

Osteoarthritis (OA) is a kind of joint degenerative disease that seriously affects patients' quality of life. Knee osteoarthritis (KOA) is the most common clinical presentation of OA, causing great damage to individuals and society [1]. The main lesions are degeneration of articular cartilage of the knee joint and secondary hyperplasia of bone. KOA mainly manifests as knee pain and limited activity [2-5]. KOA is the most common disease among the elderly, and some scholars predict that osteoarthritis represented by KOA will become the fourth most disabling disease [1]. Chinese epidemiological studies have shown the prevalence rate of KOA to be 8.1%, and the total disability rate to be 53% in females [3]. According to the Traditional Chinese Medicine’s Diagnosis and Treatment Guide for Knee Osteoarthritis (2020), KOA mainly includes five types: Qi stagnation and blood stasis, dampness and heat obstruction, cold and dampness obstruction, deficiency of liver and kidney, and qi and blood weakness [6], and KOA belongs to the category of “bone obstruction” or “Bi disease” in Traditional Chinese medicine (TCM) theories. “The Yellow Emperor's Canon of Internal Medicine” recorded that ” Osteoarthritis is not caused by the evil qi of the four seasons, the patients hate, cold and tremble; it enters the human body from the skin barrier, stays in the bones of the human, which becomes osteoarthritis.” It refers to the fact that most of the patients with KOA are physically weak. When the evil spirits come, they are weak but solid, and the evil spirits go deep into the bone marrow, leading to the disease. Diagnosis and Treatment Guide of Integrated Chinese and Western Medicine for Knee Osteoarthritis recommends the use of TCM treatment to restore some functions of patients to achieve the purpose of treating KOA [7]. A large number of studies and experiments have shown that TCM plays a significant role in the treatment of KOA. The following paper discusses in detail the role of single-herb TCM and TCM compounds in the treatment of KOA.

## 2 RELATED WORKS

We have discussed and selected the TCM monomers and compounds commonly used in the treatment of KOA and explored their main mechanism of action in the treatment of KOA through a literature search. In addition, we have studied the mechanism of action of TCM compounds and Chinese patent medicine in the treatment of KOA. Combined with the results of the literature search, we have discussed the future research direction of TCM compounds and TCM monomers in the treatment of KOA.

## 3 MATERIALS AND METHODS

Two researchers conducted a comprehensive search of four databases: China National Knowledge Infrastructure (CNKI), Wanfang Data, China Science and Technology Journal Database (VIP), and PubMed. The search was conducted up to June 2023 in each database. The four databases were searched by using subject words and free words. The following keywords were used for the literature retrieval: “knee osteoarthritis” or “osteoarthritis” and “Chinese medicine”.

## 4 SINGLE-HERB TCM FOR THE TREATMENT OF KOA

### 4.1 Mechanism of Single-herb TCM for KOA Treatment

The pathogenesis of KOA remains unclear and is mostly related to age, obesity, physical constitution, and other factors [8]. Matrix metalloproteinase 3 (MMP-3), as a stromalysin proteolytic enzyme, can lead to pathological changes, such as synovial hyperplasia and cartilage degradation, by regulating the expression of related genes. MMP-3 is believed to be closely related to the production and severity of KOA [9, 10]. Apoptosis is another cause of KOA. Autophagy, as a normal form of cell protection, is often complementary to apoptosis. Some scholars have found that the body can fight against apoptosis through autophagy, thus contributing to the relief of patients' symptoms and the repair of cartilage [11]. In addition, the occurrence of KOA is also closely related to the activation of inflammatory factors and signaling pathways [12, 13]. Researchers have confirmed that the activation of the Wnt/β-catenin signaling pathway can affect the differentiation and apoptosis of chondrocytes and then degrade cartilage, which leads to the occurrence of KOA. Researchers have found in the experimental study on human nucleus pulposus cells that inflammatory cytokine interleukin-1β (IL-lβ) regulates the expression of AdamS-4 by promoting the abnormal activation of the nuclear factor kappa-B (NF-κB) signaling pathway, resulting in the loss of aggregative Agg and type II collagen ColII [5]. As components of extracellular matrix (ECM), the loss of agglutinoglycan, Agg, and ColII can cause chondrocyte injury [14].

However, modern pharmacological studies have shown that the mechanism in the treatment of KOA-related biological processes by single-herb TCM includes the regulation of cell metabolism, apoptosis, oxidative stress response, endopeptidase activity, *etc.,* and the high-frequency signaling pathways, including tumor necrosis factor (TNF), interleukin-17 (IL-17), apoptosis, *etc*. [15]. The side effects of TCM single-herb in the treatment of KOA are less than those of the compatible drug pairs and TCM compounds, but the effect is more targeted. With the progress in TCM modernization, the active ingredients of TCM have been clarified continuously. In recent years, TCM single-herb has been widely studied because of its remarkable effect and clear composition.

### 4.2 Dipsacus Asper Wall. Ex Henry

Dipsacus asper Wall. ex Henry was first reported in the Shennong Materia Medica. It is the dried root of the perennial plant Sichuan teasel. In TCM theories, Dipsacus asper Wall. ex Henry can nourish the liver and kidney and strengthen the muscles and bones. The ancient practitioners widely used Dipsacus asper Wall. ex Henry in the treatment of orthopedic diseases; Dipsacus asper Wall. ex Henry’s Chinese name has the meaning of healing broken bones. Dipsacus asper Wall. ex Henry is often used in the treatment of lumbar muscle strain, and bone and joint diseases. The results of modern pharmacological studies have shown that it has a wide range of effects, such as antioxidant, anti-inflammatory, and bone protection, and the latter is the most characteristic pharmacological effect of Dispacus. Total saponins are its main components and also the active ingredients in the treatment of KOA. Shang *et al.* have found that total saponins can upregulate the expression levels of Atg3, Atg4, and Atg7 in the articular cartilage tissue of KOA mice, thus inhibiting the overactivation of phosphatidylinositol-3 kinase (PI3K)/threonine kinase (AKT)/mammalian target of rapamycin (mTOR) signaling pathway, and promoting the occurrence of autophagy and protecting articular cartilage [16, 17].

### 4.3 *Chaenomeles Sinensis* (Thouin) Koehne

Chaenomeles sinensis (Thouin) Koehne does not have the direct effect of nourishing the kidney and strengthening bone in the cognition of TCM doctors, but TCM doctors think that Chaenomeles sinensis (Thouin) Koehne has the effect of relaxing tendons and activating collaterals, which can improve the activity of joints and make tendons elastic. Based on the method of network pharmacology and molecular docking, 3 active ingredients of Chaenomeles sinensis (Thouin) Koehne and 44 targets related to the treatment of KOA were obtained by searching. Through GO function and KEGG pathway analysis, it was found that the common target is involved in regulating cell metabolism, apoptosis, and other biological processes. TNF, NF-κB, advanced glycation end products (AGE), receptors for advanced glycation end products (RAGE), and other signaling pathways are closely related [15, 18].

### 4.4 Rhizoma Polygoni Cuspidati

Rhizoma Polygoni Cuspidati is used to remove dampness and heat in the clinical practice of TCM theories. It is used in the treatment of KOA with the wisdom of ancient people and the exploration of modern doctors. TCM physicians believe that the onset of KOA is closely related to external evil, among which the most important evil is dampness and heat. Rhizoma Polygoni Cuspidati can not only clear dampness and heat, but also play a role in relieving pain, so it is used to relieve the symptoms of KOA patients. Pharmacological studies have shown that resveratrol is an antitoxin widely found in medicinal plants, such as Rhizoma Polygoni Cuspidati. With powerful anti-inflammatory properties, resveratrol can effectively offset the proinflammatory catabolism mediated by IL-1β, significantly improve the deposition of extraoral matrix under inflammatory conditions, and has great potential for articular cartilage repair and delay the progression of OA [19]. Relevant research results have shown that OA in obesity-related mice was induced by a high-fat diet and resveratrol treatment was found to be able to effectively reduce the level of excess autophagy caused by obesity and improve the pathological changes of obesity-related OA [20]. In addition, some researchers have injected resveratrol locally into the articular cavity of OA mouse models. They have found that the intra-articular injection of resveratrol can delay the degeneration of articular cartilage, and further proved that this may be related to the regulation of adenosine 5‘-monophosphate (AMP)-activated protein kinase (AMPK)/mTOR signaling pathway and the promotion of chondrocyte autophagy [21].

### 4.5 Achyranthis Bidentatae Radix

Achyranthis Bidentatae Radix is known for its resemblance to the knee of an ox. The TCM doctors believe that it has the effect of “complementing the shape “, so it is often used to treat Bi disease. Phytochemical and pharmacological studies have shown that Achyranthes bidentata polysaccharides (ABPS) are the main active ingredients of Achyranthis Bidentatae Radix in treating KOA. By promoting Beclin-1 and microtubule-associated protein1 light chain 3-II (LC3-II)/I protein expression in chondrocytes, ABPS can reduce the expression of Fas-associating protein with a novel death domain (Fadd), improve the autophagy level in chondrocytes of OA, and reduce the apoptosis of chondrocytes. In addition, basic studies have shown that it can effectively inhibit the activation of caspase-3, downregulate the proapoptotic proteins Bad and Bax, and upregulate the expression level of the antiapoptotic protein Bcl-xL. It can also inhibit the phosphorylation of p53 protein to inhibit IL-1β-induced apoptosis and protect chondrocytes [22, 23].

### 4.6 Other Single-herb TCM

Eucommia ulmoides Oliv. is commonly used for bone injury in TCM, which can tonify the liver and kidney, strengthen muscles and bones, and prevent miscarriage. The water extract of Eucommia ulmoides Oliv. may delay cartilage degeneration by inhibiting the PI3K/AKT pathway, reducing the expression of inflammatory factors IL-1β and IL-6, inhibiting the secretion of MMP-3, downregulating phosphorylated AKT, and inhibiting the progress of KOA [24, 25].

Paeoniflorin is the main pharmacodynamic component of Paeoniae Radix Alba. Researchers have found that the autophagy level of chondrocytes decreased after IL-1β treatment, but after the intervention of paeoniflorin, the PI3K/AKT signaling pathway was inhibited. LC3-II and Beclin-1 expression levels in chondrocytes were increased, autophagy activity was increased, and the concentration level of inflammatory factors was decreased at the same time. Paeoniflorin could effectively relieve the damage of chondrocytes [26].

Angelica sinensis was first reported in Shennong Materia Medica. It is one of the main TCM monomers for promoting blood circulation. It also has the effect of promoting ventilation, dispelling evil wind, and treating the Bi disease. Some studies have shown that angelica polysaccharide can protect chondrocytes from hydrogen peroxidation-induced oxidative stress and subsequent apoptosis through its antioxidant and anti-inflammatory effects. Some studies have shown angelica polysaccharide to activate autophagy in chondrocytes through the extracellular signal-regulated kinase 1/2 (ERK1/2) signaling pathway and protect chondrocytes from apoptosis [27, 28].

Curcumin is the most active component in the rhizome of Curcuma longa L, showing good therapeutic effects on a series of chronic inflammation-related diseases. Curcumin has been proven to have reliable antioxidative stress, anti-apoptosis, and anti-catabolic effects in the relevant studies on OA treatment. Some researchers have confirmed in *in vitro* experiments that curcumin can increase the expression of autophagy-related proteins Beclin-1 and LC3-II, induce chondrocyte autophagy to inhibit apoptosis and inflammatory signal transmission, promote chondrocyte repair and proliferation, and play a protective role in cartilage [29, 30].

## 5 THE TREATMENT OF KOA BY CHINESE HERBAL COMPOUNDS

### 5.1 Mechanism of Action of Chinese Herbal Compounds in KOA

TCM compounds refer to a prescription that is composed of two or more TCM monomers, and involves the corresponding processing method and use. TCM compounds are designed for relatively definite disease syndromes. The combinatorial criteria of four Qi and five Wei can greatly improve clinical efficacy. All TCM compounds include a ruler, minister, assistant, and guide. For example, the rulers and ministers play a dominant role in the treatment of KOA. Therefore, rulers and ministers have the functions of ” nourishing kidney”, “strengthening bone” and “promoting collaterals” of TCM theories. TCM compounds aim to treat KOA from the root and the adjuvants are flexible, which can treat secondary symptoms and reduce the toxicity of the main drugs of the ruler and minister. For example, some doctors tend to add spleen-nourishing drugs into the treatment, because the spleen is Houtian zhiben and the kidney is Xiantian zhiben, and the two can complement each other. Assistants and guides are used to mix various drugs or introduce drugs into the meridian so that the combination of multiple TCM monomers can work together, especially in slowing the progress of KOA.

### 5.2 Yougui Wan

Yougui Wan (YW), from the Complete Compendium of Zhang Jingyue, has the effect of tonifying kidney Yang, which has a functional meaning in the understanding of TCM. According to TCM theories, tonifying the kidney Yang can enhance the function of the kidney and thus achieve the purpose of strengthening the bones. The studies have shown the TNF signaling pathway to be significantly expressed after YW treatment. TNF is a proinflammatory cytokine that can activate inflammatory cytokines, such as IL-1β, IL-6, and TNF-α, as well as MMP genes, such as MMP-3, MMP-9, and MMP-13. It can regulate joint local inflammation and cartilage tissue remodeling. A study has shown that YW can not only regulate inflammation, but also inhibit the degradation of articular cartilage, thus exerting a therapeutic effect on KOA. At the same time, YW can inhibit the expression of IL-1, IL-6, and TNF-α, reduce their serum levels, and alleviate the inflammatory response [31]. Relevant randomized controlled trials (RCTs) have also shown that after the intervention of the rat model YW, the articular cartilage edges of the low-dose and medium-dose YW groups were uneven and the arrangement of chondrocytes was disordered. The cartilage structure of the YW high-dose group tended to be normal [32].

### 5.3 Duhuo Jisheng Decoction

Duhuo Jisheng Decoction (DJD) is used to treat KOA in the clinic, mainly for treating Bi disease for a long time and deficiency of the liver and kidney. TCM doctors believe that there are mainly three kinds of external evil qi causing KOA, wind, cold, and wet, but the three evils are intermixed with each other to cause the disease, and DJD can not only remove cold and wet, but can also tonify liver and kidney, thereby playing a solid role in KOA's decline. Modern pharmacology has shown that DJD can reduce damage to joints and cartilage by regulating the expression level of inflammatory cytokines in KOA patients and inhibiting the inflammatory response. Relevant studies have shown that DJD can inhibit the release of IL-1 in the joint fluid of KOA patients, reduce the body's inflammatory response, and relieve joint pain [33]. DJD was used to treat 35 patients with KOA, and the contents of IL-1, TNF-α, and OA index scores in the joint fluid of patients were significantly decreased after treatment (*p*<0.05). It has been found that DJD could significantly reduce the contents of inflammatory cytokines TNF-α, IL-1β, and hypersensitive C-reactive protein (hs-CRP) in the synovial fluid of the knee joint in KOA patients (*p*<0.05), and its efficacy was comparable to that of meloxicam. Wang Huang *et al.* have conducted an experimental study on animal model rats and detected the contents of IL-1β and IL-8 in the synovium of rats by immunohistochemical method, and found that the contents of IL-1β, and IL-8 in the synovium of rats after the intervention of DJD were significantly decreased compared to that before treatment. The arthritis index score was significantly decreased compared to that before the treatment (*p*<0.05) [34]. The researchers have confirmed that DJD could reduce the inflammatory response of knee synovium and delay cartilage degeneration by inhibiting the release of inflammatory factors, such as TNF-α, IL-1β, and hs-CRP in KOA patients. Meanwhile, studies have shown that transforming growth factor-β (TGF-β) and IL-1 can regulate the release of matrix metalloproteinases (MMPs) and other proteolytic enzymes during the pathogenesis of KOA, inducing the degradation of KOA and articular cartilage [33-35].

### 5.4 Simiao Wan

Simiao Wan (SW) contains only four TCM monomers, which play a role in removing dampness and heat in TCM. In view of TCM theories, evil spirits entering the body for a long time will turn into heat, so some patients of KOA show clinical manifestations of dampness and heat. At this time, SW can remove dampness and heat, thus making the meridians smooth, and qi and blood to flow smoothly. Some researchers have treated the rat model with SW, and the results have shown that the expression of LC3 in chondrocytes was increased after Simiao powder treatment, while the expressions of Fadd and caspase-3 were decreased; the progression of rat osteoarthritis was also relieved. It is suggested that the apoptosis of chondrocytes may be inhibited by the autophagy of chondrocytes activated by the SW [36]. Relevant RCTs have shown that SW can significantly reduce the expression of IL-1β and TNF-α, effectively inhibit the inflammatory response, and also effectively inhibit the inflammatory response in the pathological process of KOA [37].

### 5.5 Other Curative TCM Compounds

In recent years, the involvement of TCM compounds in the regulation of chondrocyte autophagy has become a hot topic. Clinical studies have observed that compared to conventional Western medicine treatment, the expression of chondroautophagy-related protein Beclin-1 in patients treated with Peony and Shaoyao Gancao Fuzi Tang (SGFT) increased. The expressions of PI3K, pAkt, and mTOR decreased, the level of chondrocyte autophagy increased, and the related symptoms of patients with osteoarthritis were more significantly relieved than those in the Western medicine treatment group [38]. Some researchers have found that Bushen Huoxue Fang (BHF) could effectively relieve pain and improve joint function scores in KOA patients in controlled clinical trials. Subsequently, animal experiments have shown that BHF could effectively reduce the serum inflammation level of KOA mice and reduce the degradation of the extracchondral matrix. Its mechanism may be related to the activation of autophagy in cartilage and the inhibition of related inflammatory signaling pathways [39].

At the same time, researchers have prepared the KOA chondrocyte model through IL-1β induction. They have found that it could effectively downregulate the levels of inflammatory factors IL-6, TNF-α, and MMP-13 in the KOA chondrocyte model and upregulate the expression of key autophagy factors in chondrocytes after the intervention of Jiawei Yang He Tang (JYHT) medicated serum. It reflects that Jiawei Yanghe decoction can regulate the autophagy level of chondrocytes and reduce the level of inflammation. It plays a protective role in chondrocytes. Kong Tian *et al.* have found that although nonsteroidal anti-inflammatory drugs (NSAIDs) alone could relieve the symptoms of the modeling mice, they did not affect the autophagy level of chondrocytes. However, after Yangxue Yishen Fang (YYF) was added, the expression level of LC3 in the cartilage of the modeling mice was increased and the symptom improvement effect was better than that of Western medicine. This suggested that YYF could play a protective role in articular cartilage by regulating chondrocyte autophagy [14]. The above research results have confirmed that many classic TCM compounds can achieve a protective effect on articular cartilage by affecting autophagy.

### 5.6 Treatment Modes of TCM Compounds

#### 5.6.1 Study on the Treatment of KOA by External Application of TCM Compounds

Chinese herbal paste, fumigation, hot ironing, the penetration effect of drugs, and heat energy after heating the affected area, play the role of warming the meridian and dispersing evil, thereby promoting blood circulation and clearing collage. They can improve local blood circulation and metabolism, promote lymphatic reflux, and accelerate the dissipation of inflammatory response. Combined with the physical effects of TCM monomers or compounds, acupoints, meridians, and currents, the ion import method of TCM monomers or compounds can enhance the transdermal absorption of herbal medicine and stimulate the acupoints to relieve pain. Clinical TCM therapy is often combined with joint cavity sodium hyaluronate injection, arthroscopic surgery, and other therapies to improve joint pain and joint function, thus improving clinical efficacy. Compared to the treatment of non-steroidal anti-inflammatory and analgesic drugs, the integrated treatment of TCM and Western medicine has the advantages of targeting the etiology, directly acting on the local joint, simple operation, and few systemic toxic and side effects. It can also be combined with acupuncture, massage, moxibustion, and other external treatments to relieve patients' pain and other symptoms [6].

#### 5.6.2 Progress in the Treatment of KOA with Chinese Patent Medicine

Compared to TCM monomers and TCM compounds, Chinese patent medicine is convenient to carry and easy to promote. There are various kinds of Chinese patent medicines to treat KOA, which are stable in form and easy to take. With the development of TCM technology, there are many Chinese patent medicines to treat KOA, such as Wangbi tablets, Xianling Gubao capsules, DuhuoJisheng pills, Jintiange capsules, Longbie capsules, *etc*. These medicines have a remarkable effect on KOA. Studies have shown that Longbie capsules (LBs) can eliminate the knee swelling of KOA rats and effectively treat the clinical symptoms of KOA, and their mechanism of action is related to the reduction of serum IL-1β and IL-6 levels and an increase in IL-10 levels in KOA rats [40]. Xianling Gubao capsules (XGs) inhibited bone resorption; XGs could significantly reduce the number of osteoclasts and the content of N-telopeptide of type I collagen (NTX), a specific marker of bone resorption, and significantly increase the bone mineral density (BMD) and bone trabecular volume in rats. It has been suggested that this effect may be related to decreasing bone resorption and increasing bone formation. Some studies have also shown that xanthoxylin can increase the content of bone morphogenetic protein 2 (BMP-2) and osteocalcin (OC), thus promoting bone formation and inhibiting bone resorption in osteoporosis rats. It has been reported that XGs could increase the BMD of rabbits in an arthritis model, improve the bone trabecular structure, upregulate the content of BMP-2, downregulate the content of MMP-13, and play an anti-osteoarthritis role [41].

## 6 DISCUSSION

TCM monomers or TCM compounds can treat KOA or delay the progression of the disease by regulating signaling pathways, reducing the content of matrix metalloproteinases, and regulating autophagy. Many studies have shown the effectiveness of TCM monomers and TCM compounds in the treatment of KOA. Compared to Western medicine, TCM monomers or TCM compounds have the advantages of fewer side effects, fewer adverse reaction reports, more diverse treatment techniques, and higher short-term treatment satisfaction. Most TCM monomers and compounds have multitarget and multi-pathway effects, so compared to Western medicine, they have higher drug resistance, and the therapeutic effect is less likely to decay over time.

However, the study on the treatment of KOA by TCM monomers and TCM compounds also has certain limitations and deficiencies. Firstly, the substances in TCM monomers and TCM compounds are relatively complex, and the content of active ingredients is difficult to monitor. It may lead to the failure of the optimal therapeutic concentration of active ingredients in the original TCM decoction methods, thus failing to exhibit the best therapeutic effect. Secondly, the research on TCM monomers and TCM compounds is still in the initial stage. At present, the basic research on TCM treatment of KOA is mostly based on the NF-κB, MAPK, PI3K/AKT, and Wnt/β-catenin pathways, and the discussion of individual target proteins. However, there is little research on TGF-β and toll-like receptor signaling pathways and axin. Thirdly, studies on the cytotoxicity and bioavailability of TCM are lacking. There is also a lack of research on the difference between external application and oral effect of the same TCM monomers or compounds, and the best way of different TCM monomers or compounds for treating KOA is unclear. Fourthly, the communication of research data is also limited, and the data obtained by using different databases may be different. All these limitations postpone the clinical application of TCM in the treatment of KOA. In addition, there are still problems with whether the active ingredients of the TCM monomers are consistent with those of the compound after formulation, or whether there are new active ingredients that do not exist in the monomers in the preparation of the compound.

Therefore, it is urgent to further strengthen the combination of TCM and modern pharmacological research, and further dig out the effective components of TCM in the treatment of KOA as well as the specific changes in the active components of each TCM monomer in the compound. We should strengthen the construction of databases and platforms, and connect them with international data platforms [15]. In the future, more extensive and advanced technologies should be used to fully explore the pharmacological effects of TCM monomers or compounds. A large sample, multi-center basic research, and clinical research should be carried out to improve the credibility of TCM treatment of KOA and further improve the clinical efficacy of TCM. At the same time, the potential therapeutic effect of the active ingredients on KOA is the result of the comprehensive action of multiple pathways and multiple targets. Future studies should actively utilize modern pharmacological means to deeply and accurately understand the mechanism of action and the target of action, and screen out the effective monomer components, to accumulate effective and more widely used candidate drugs for the treatment of KOA. The changes in metabolites before and after KOA treatment by using TCM monomers or TCM compounds that have been verified to have exact clinical efficacy should also be studied. The whole pathogenesis of KOA and the intervention process of drugs can be monitored by using the research methods of metabonomics. Differential metabolites from endogenous metabolites can be found to provide direction for the early diagnosis of KOA. This may also provide a modern and international basis for TCM treatment of KOA [42].

In conclusion, TCM monomers and TCM compounds are effective in treating KOA and can make certain contributions to modern medical treatment in clinical practice. It may be an important direction for future research to combine the study of the pathogenesis of KOA with the study of effective active ingredients [43]. TCM monomers and TCM compounds that can be used in the treatment of KOA should be evaluated with a more in-depth combination of research methods, such as network pharmacology and metabolomics, and more attention should be paid to the accurate use of TCM to regulate the course and development of KOA. This will contribute to the modernization of TCM and the study of pathology and the physiology of KOA [21, 44].

## CONCLUSION

The combination of TCM and various forms of treatment provides the means of treating KOA in a more diversified and flexible manner, so that patients can have more choices. With the continuous progress of technology, in-depth studies have been conducted on the incidence and progress of TCM monomers and TCM compounds in the treatment of KOA. It has been confirmed that TCM has a certain curative effect on the treatment of KOA, which makes more patients accept TCM treatment. However, it is urgent to further study the pharmacology of the mechanism of action of TCM monomers and the pharmacology of the active ingredients of TCM compounds, especially the pharmacology of Chinese patent medicine, so as to help the majority of patients. In addition, the pharmacology of the external use of TCM monomers or TCM compounds, external use of TCM monomers or TCM compounds combined with other external therapies, or internal administration of TCM monomers or TCM compounds combined with external use, needs to be studied. Their influence on the specific incidence and progression of KOA also needs further study. With the deepening of the research on the pathogenesis of KOA and the updating of research methods, TCM is sure to play a greater role in the treatment of KOA. In conclusion, TCM monomers and TCM compounds can have certain positive effects on the onset and progression of KOA, which can help in reducing the pain of the patients in clinical treatment and delay the time of surgical treatment.

## FUTURE WORK

In future studies, we will focus more on the limitations of research techniques related to TCM monomers and TCM compounds, and compare the advantages of research techniques in different studies. Our study has lacked further discussion on the mechanism of action of proprietary Chinese patent medicines, which we will continue to explore in subsequent studies.

## LIST OF ABBREVIATIONS

BHF: Bushen Huoxue Fang
BMD: Bone Mineral Density
BMP-2: Bone Morphogenetic Protein 2
CNKI: China National Knowledge Infrastructure
DJD: Duhuo Jisheng Decoction
JYHT: Jiawei Yang He Tang
KOA: Knee Osteoarthritis
LBs: Longbie Capsules
NSAIDs: Nonsteroidal Anti-inflammatory Drugs
OA: Osteoarthritis
OC: Osteocalcin
RCTs: Randomized Controlled Trials
RAGE: Receptors for Advanced Glycation end Products
SGFT: Shaoyao Gancao Fuzi Tang
TCM: Traditional Chinese Medicine
TNF: Tumor Necrosis Factor
XGs: Xianling Gubao Capsules
YW: Yougui Wan
YYF: Yangxue Yishen Fang
